# Pleomorphic xanthoastrocytoma in the multiverse of epigenomics: is it time to recognize the variants?

**DOI:** 10.1186/s40478-022-01390-z

**Published:** 2022-06-07

**Authors:** João Víctor Alves de Castro, Felipe D’Almeida Costa

**Affiliations:** Department of Anatomic Pathology, AC Camargo Cancer Center, São Paulo, SP Brazil

**Keywords:** Methylation analysis, Epigenetics, Glioma, Pleomorphic xanthoastrocytoma, Morphology, Tumor classification

## Main text

Historically, the designation of “Pleomorphic xanthoastrocytoma” (PXA) arose when a glial nature was ascribed to mostly superficial lesions with frequent leptomeningeal involvement and morphologic features considered to belong to a spectrum of “monstrocellular sarcomas” and Glioblastomas that nevertheless showed indolent behavior [1]. Although strikingly bizarre cells were consistently present in these lesions, the lack of necrosis and microvascular proliferation set this group of lesions apart from bona fide Glioblastomas. Conversely, although the presence of lipidized cells in the central nervous system was frequently considered to include xanthomas in the differential diagnosis, the discovery of glial fibrillary acidic protein (GFAP) as a marker of glial lineage was paramount to establish the true nature of PXA. Further studies with electron microscopy also provided a rationale for the frequent reticulin deposition around individual cells by showing basement membrane material around neoplastic cells, a feature not only seen in mesenchymal lineage cells but also in a subset of subpial astrocytes [1]. This constellation of findings led to the recognition of PXA as a distinct entity since the 1993 WHO Classification of Brain Tumors [2].

Besides the recognition of indolent PXA cases, a subset of mitotically active PXA was recognized to be clinically aggressive. Apart from a higher mitotic index, such cases displayed more uniform cells with epithelioid features, loss of reticulin staining around individual cells, and rare or even absent eosinophilic granular bodies [3]. This prompted a morphological overlap with a rare Glioblastoma variant first described by Rosenblum et al. in 1993, so-called “Lipid-rich epithelioid glioblastoma” [4]. Critically, not only a morphologic but also a clinical overlap was noted, with both types behaving in an aggressive fashion, with intermediate prognosis between the prototypical Glioblastoma and PXA with anaplastic features.

Recently, most molecular studies including cases of PXA and Epithelioid glioblastomas underscore this overlap between two seemingly identical entities on morphological grounds alone [5, 6]. Most importantly, not only the so-called “Epithelioid glioblastomas'' show morphological overlap with PXA, but also tumors with features of otherwise conventional Glioblastomas, Astroblastomas [7], Gangliogliomas, non-specific low grade diffuse gliomas and even spindle-cell or embryonal-like [8] lesions have been assigned to the methylation class PXA [9]. Besides the epigenomic similarity, these phenotypically highly variable tumors also demonstrate a copy number variation profile and mutational signatures most consistent with PXA (high frequency of *CDKN2A/B* homozygous deletion and most often *BRAF* V600E but also other MAPK activating alterations) [9].

In a recent paper by Ebrahimi et al. published in this journal, the authors discussed the challenges associated with the diagnosis and grading of PXA using morphologic and molecular criteria, especially including methylation profiling [10]. Their investigation of histologically defined PXA (histPXA) and PXA defined by DNA methylation analysis (mcPXA) highlights the wide spectrum of morphological patterns that fall under mcPXA and further explores its distinction from aggressive entities with similar morphology, such as Glioblastoma, IDH-wildtype (GBM, IDH-wt) using methylation profiling studies.

Astonishingly, only around one third of epigenetically defined PXA had the classic morphology associated with this entity. This implies that, using the current CNS WHO definition, more than half of PXA cases would be missed by a lack of histopathological consideration of this differential diagnosis. In addition, more than half of histPXA cases did not fall into the mcPXA, reinforcing several other tumor entities may show PXA-like histological features [10].

**Fig. 1:**
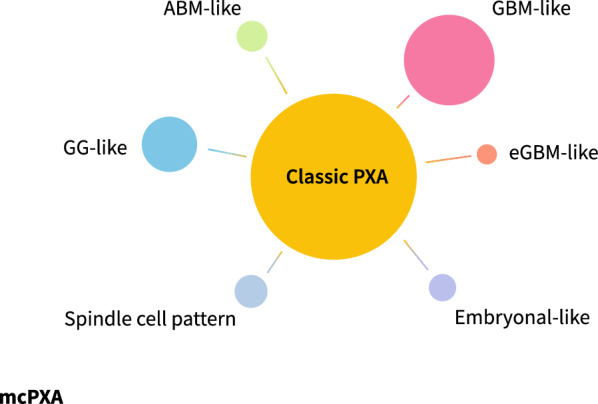
Morphological spectrum of epigenetically defined PXA (mcPXA)

Taking such robust data into consideration with previous challenges we encountered in our practice, we propose a systematic recognition of histologically and molecularly defined PXA cases and its morphologic variants, hoping to reduce pitfalls related to ancillary testing and final integrated report (Table 1 and Fig. 1).

**Table 1:** Proposed morphological variants of PXA

Morphological pattern	Histological description	Differential diagnosis
Classic	Variably pleomorphic cells with eosinophilic cytoplasm, areas of spindling and fascicular growth, and at least focal lipidized neoplastic cells with EGBs	Ganglioglioma, Pilocytic astrocytoma, Giant cell GBM
Ganglioglioma-like	Compact and fasciculated tumor with ganglion cell differentiation and EGBs	Ganglioglioma and other low-grade glial neoplasms with ganglion cell differentiation
Glioblastoma-like	Features resembling conventional glioblastoma, including mitotically active tumor with pseudopalisading necrosis and microvascular proliferation	Conventional GBM IDH-wt and HGG IDH/H3-wt
Epithelioid glioblastoma-like	Predominantly epithelioid cells with distinct cell borders and round nuclei with prominent nucleoli arranged in a solid pattern	GBM IDH-wt and HGG IDH/H3-wt with epithelioid features: non-flat copy-number profile, including gene amplifications (*CDK4, CDK6, EGFR, MDM2, MDM4, MET*)
Astroblastoma-like	Astroblastic rosettes might be found at least focally in tumors with areas of classic PXA and absence of *MN1* fusions	Astroblastoma (ABM): presence of *MN1* alterations
Spindle cell	Spindle cell pattern in the majority or entirety of the specimen	Meningothelial and mesenchymal, non-meningothelial neoplasms
Embryonal-like	Monomorphic undifferentiated and/or rhabdoid cells reminiscent of true embryonal neoplasms, including INI-1 (*SMARCB1*) loss	Atypical teratoid/rhabdoid tumor and other Embryonal tumors

This broad morphological spectrum highlights useful criteria to guide pathologists in the consideration of PXA as a differential diagnosis whenever facing a tumor with morphological patterns diverse than first appreciated by Kepes et al*.* in 1973.We stress the major role of methylation profiling and CNV analysis in precisely classifying gliomas with concomitant activating MAPK alterations/*CDKN2A/B* deletion in the setting of a neoplasm without classic PXA morphology, hoping to improve consistency in the diagnosis as a means to ensure proper diagnostic workup and treatment of such lesions.

Whether DNA methylation, other molecular techniques or histopathology should be the cornerstone for tumor classification is a matter of ongoing discussion. Specific molecular alterations have already been used to define CNS tumor entities, which are included in the new WHO blue book. For diffuse gliomas located in midline sites, the *H3-3A* K27M mutation establishes the diagnosis of diffuse midline glioma, H3 K27-altered, irrespective of tumor morphology. The *H3-3A* G34R/V mutations in supratentorial infiltrating gliomas define a Diffuse hemispheric glioma, H3 G34-mutant, with a broad morphological spectrum, ranging from glioblastoma-like to embryonal-like. Taking these tumor types as examples, it seems to be reasonable to use a genome-wide methylation profile to improve our understanding of PXA, expanding its possible histopathological presentations. On the other hand, from the clinicians’ perspective, it may be tempting to shift to a model of classification that considers only the molecular alterations, but this approach may be misleading, because finding a single genetic alteration, out of the context of a specific tumor entity, may not be predictive of treatment response for every patient.

For example, *BRAF* V600E can be found in supratentorial Pilocytic astrocytoma (PA), Ganglioglioma (GG), Pleomorphic xanthoastrocytoma (PXA), some cases of High grade astrocytoma with piloid features (HGAP) and true Glioblastoma, IDH-wildtype (GBM), the latter both with and without epithelioid morphology. This shared mutation does not mean they will respond equally to anti-BRAF therapies, probably due to the additional molecular alterations they possess. While PA and GG usually have only a MAPK alteration, PXA commonly show a MAPK alteration together with *CDKN2A/B* loss, HGAP is a combination of MAPK, *CDKN2A/B* loss and a telomere maintenance mechanism (usually through *ATRX* mutation but also rare *TERT* promoter mutations) and GBM frequently shows the + 7/− 10 signature and oncogene amplifications. This constellation of genetic alterations usually demands more than a single and simple molecular test to be found, so the whole landscape of the tumor biology may not be fully appreciated at the time of diagnosis, leading to unexpected clinical outcomes and tumor responses.

In our point of view, the best diagnostic approach is still the integrated diagnosis method proposed since the Haarlem consensus, published in 2014 and adopted by WHO classification in 2016 and 2021. The integrated diagnosis, as its name implies, incorporates all available data, from morphology to immunohistochemistry, genetics and epigenetics, in order to reach a more accurate classification. It has the potential for a precise diagnosis, appropriate treatment choice and patient selection for targeted therapies and clinical trial enrollment. In summary, the growing knowledge of the molecular aspects of the CNS neoplasms need to be used not in substitution of morphology, but in a complementary fashion, sometimes conducting pathologists to reassess and improve our histopathological evaluation and tumor classification.

## Data Availability

No data was generated.

## Abbreviations

PXA: Pleomorphic xanthoastrocytoma
GFAP: Glial fibrillary acidic protein
histPXA: Pleomorphic xanthoastrocytoma, histologically defined
mcPXA: Methylation class Pleomorphic xanthoastrocytoma
GBM, IDH-wt: Glioblastoma, isocitrate dehydrogenase-wildtype
MAPK: Mitogen-activated protein kinase
PA: Pilocytic astrocytoma
GG: Ganglioglioma
HGAP: High grade astrocytoma with piloid features
